# Etiology and Selected Multidrug-Resistance Patterns in Urinary Bacterial Isolates: A Comparative Analysis of Outpatients and Inpatients at a Tertiary Hospital in Mexico City

**DOI:** 10.3390/microorganisms14091946

**Published:** 2026-09-02

**Authors:** Vladimir Paredes-Cervantes, Cecilia Rosel-Pech, Sandra Angélica Rojas-Osornio, Edith Reyes-Serrato, Laura López-Pelcastre, Leticia Manuel-Apolinar, Laura Arcelia Montiel-Cervantes, José Molina-López, María Pilar Cruz-Domínguez, José Guadalupe Rendón-Maldonado, Fernando Minauro-Sanmiguel, Martha Eugenia Ruiz-Tachiquín, Salvador Vázquez-Vega, Emiliano Tesoro-Cruz

**Affiliations:** 1 Laboratorio Central, Hospital de Especialidades, Centro Médico Nacional “La Raza”, Instituto Mexicano del Seguro Social, Mexico City 02990, Mexico; vladimir.paredes@imss.gob.mx (V.P.-C.); rese8228@gmail.com (E.R.-S.); laura.lopezp@imss.gob.mx (L.L.-P.); 2 Hospital de Psiquiatría “Morelos”, Instituto Mexicano del Seguro Social, Mexico City 07450, Mexico; ceci.roselp@gmail.com; 3 Departamento de Formación Básica Disciplinaria, Academia de Bioquimica Medica II, Escuela Superior de Medicina, Instituto Politécnico Nacional, Mexico City 11340, Mexico; sandii38@yahoo.com.mx; 4 Unidad de Investigación Médica en Enfermedades Endocrinas, Hospital de Especialidades, Centro Médico Nacional Siglo XXI, Instituto Mexicano del Seguro Social, Mexico City 06720, Mexico; leticia.manuel@imss.gob.mx; 5 Unidad de Investigación en Medicina Traslacional en Enfermedades Hemato Oncológicas, Centro Médico Nacional “La Raza”, Instituto Mexicano del Seguro Social, Mexico City 02990, Mexico; laura.montielc@imss.gob.mx; 6 Unidad Periférica de Investigación Básica y Clínica en Enfermedades Infecciosas, Departamento de Salud Pública, División de Investigación, Facultad de Medicina, Universidad Nacional Autónoma de Mexico, Mexico City 04510, Mexico; joseml@unam.mx; 7 Laboratorio de Patogenicidad Bacteriana, Unidad de Hemato-Oncología e Investigación, Hospital Infantil de México Federico Gómez, Facultad de Medicina, Universidad Nacional Autónoma de Mexico, Mexico City 04510, Mexico; 8 Dirección de Educación e Investigación en Salud, Hospital de Especialidades “Dr. Antonio Fraga Mouret”, Centro Médico Nacional “La Raza”, Instituto Mexicano del Seguro Social, Mexico City 02990, Mexico; maria.cruzdo@imss.gob.mx; 9 Posgrado en Ciencias Biomédicas, Facultad de Ciencias Químico-Biológicas, Universidad Autónoma de Sinaloa, Culiacán 80010, Mexico; jgrendonm@uas.edu.mx; 10 Unidad de Investigación Médica en Genética Humana, Centro Médico Nacional Siglo XXI, Instituto Mexicano del Seguro Social, Mexico City 06720, Mexico; misaf73@yahoo.com.mx; 11 Unidad de Investigación Médica en Enfermedades Oncológicas, Unidad Médica de Alta Especialidad, Hospital de Oncología, Centro Médico Nacional Siglo XXI, Instituto Mexicano del Seguro Social, Mexico City 06720, Mexico; mertachiquin@gmail.com; 12 Unidad de Investigación Epidemiológica y en Servicios de Salud, Centro Médico Nacional Siglo XXI, Instituto Mexicano del Seguro Social, Mexico City 06720, Mexico; 13 Unidad de Investigación Biomédica en Inmunología e Infectología, Hospital de Infectología, Centro Médico Nacional “La Raza”, Instituto Mexicano del Seguro Social, Mexico City 02990, Mexico

**Keywords:** bacteria, urinary tract infections, urinary bacterial isolates, antibiotics, multidrug resistance, multidrug-resistance patterns, outpatients, inpatients

## Abstract

Antimicrobial resistance poses a critical challenge to global public health. Urinary tract infections are one of the leading causes of morbidity in Mexico. This study aimed to identify the most prevalent bacterial pathogens in urinary bacterial isolates and to determine their antimicrobial and selected multidrug-resistance pattern profiles in outpatient and inpatient isolates at a tertiary care hospital in Mexico City. In this retrospective laboratory-based surveillance study, a census of 3434 urine samples collected between January and December 2023 was analyzed. Bacterial identification and antimicrobial susceptibility testing were performed using a VITEK 2 XL automated system. *Escherichia coli* was the predominant uropathogen in both groups, followed by *Enterococcus* spp. and *Klebsiella* spp. A significant disparity was observed in the resistance profiles: *E. coli* resistance to third-generation cephalosporins and fluoroquinolones was higher in inpatient isolates than in outpatient isolates. The selected multidrug-resistance patterns were generally higher in the inpatient isolates than in the outpatient isolates. Although common enteric pathogens with lower resistance levels predominated in the outpatient isolates, inpatient isolates required coverage targeting higher resistance. These findings reflect the need to tailor the treatment to the patients’ clinical context, launch awareness campaigns for the strategic use of antibiotics, and strengthen epidemiological surveillance.

## 1. Introduction

Antimicrobial resistance (AMR) has become a major public health challenge worldwide. In 2019, AMR directly caused 1.27 million deaths worldwide. It has contributed to an additional 4.95 million deaths. It is estimated that by 2050, the annual number of deaths attributable to AMR could reach 10 million if insufficient action is taken to control it [1]. An increase in AMR also increases the economic costs of healthcare systems [2]. Globally, urinary tract infections (UTIs) are among the most common infections and affect approximately 150 million people annually [3]. These infections range from cystitis to pyelonephritis and tend to be recurrent [4].

Differences in the etiological agents of UTIs have been previously observed. *Escherichia coli* is the most common causative agent in community and inpatient settings [4].

However, other Gram-negative pathogens, such as *Klebsiella*, *Enterobacter*, and *Pseudomonas*, and Gram-positive ones, such as *Staphylococcus aureus* and *Enterococcus*, are significantly observed among inpatients [5].

According to the US National Institutes of Health, *Enterococcus faecium*, *S. aureus*, *Klebsiella pneumoniae*, *Acinetobacter baumannii*, *Pseudomonas aeruginosa*, and various enterobacteria are the primary causes of nosocomial infections worldwide, most of which exhibit the highest rates of severe AMR [6]. In general, the causative agents of hospital-acquired UTIs exhibit higher AMR rates than those of community-acquired UTIs (67.7% vs. 49.4%) [7,8,9].

In Mexico, UTIs rank third among the causes of morbidity, surpassed by respiratory and gastrointestinal infections [10]. Previous studies have reported up to 61.0% of AMR bacteria isolated from UTIs in hospitals [11]. In the specific case of *E. coli*, nearly 70.0% of the hospital isolates analyzed were AMR [12], whereas in community-acquired UTI isolates, 34.0% of the *E. coli* isolates produced extended-spectrum beta-lactamases (ESBLs) [13].

Specific health policies have been implemented to systematically collect and analyze information on the incidence of hospital-acquired infections and antimicrobial use in Mexico. These include the Official Mexican Standard NOM-045-SSA2-2005 [14] and the “Action Plan Against Antimicrobial Resistance for Mexico” [15]. These measures also help track the evolution of AMR.

Current evidence from Mexico provides limited comparative information on how bacterial etiology and multidrug resistance differ between community- and hospital-associated urinary tract infections within the same healthcare setting. This could limit the development of context-specific treatment recommendations. In this study, we aimed to characterize and compare the etiological spectrum, antimicrobial susceptibility, and selected multidrug-resistance (MDR) patterns of uropathogens isolated from outpatients and inpatients attending a tertiary care hospital in Mexico City, generating locally relevant evidence to inform antimicrobial stewardship strategies.

## 2. Results

### 2.1. Sociodemographic and Clinical Characteristics

A total of 3434 urine samples were analyzed from outpatients and inpatients. Outpatients accounted for a higher proportion in the age groups compared with inpatients (*p* = 0.022). The largest age group consisted of young adults aged 18–54 years as outpatients. A predominance of females was observed among outpatients, with a predominance of male inpatients (*p* = 0.012). Most outpatients were referred from the urology, nephrology, or kidney transplantation units. Among hospitalized patients, the departments with the highest number of cases were internal medicine, urology, nephrology, neurology, intensive care unit, and cardiology (*p* < 0.001) (Table 1).

### 2.2. Identified Uropathogenic Bacteria

**Figure 1:**
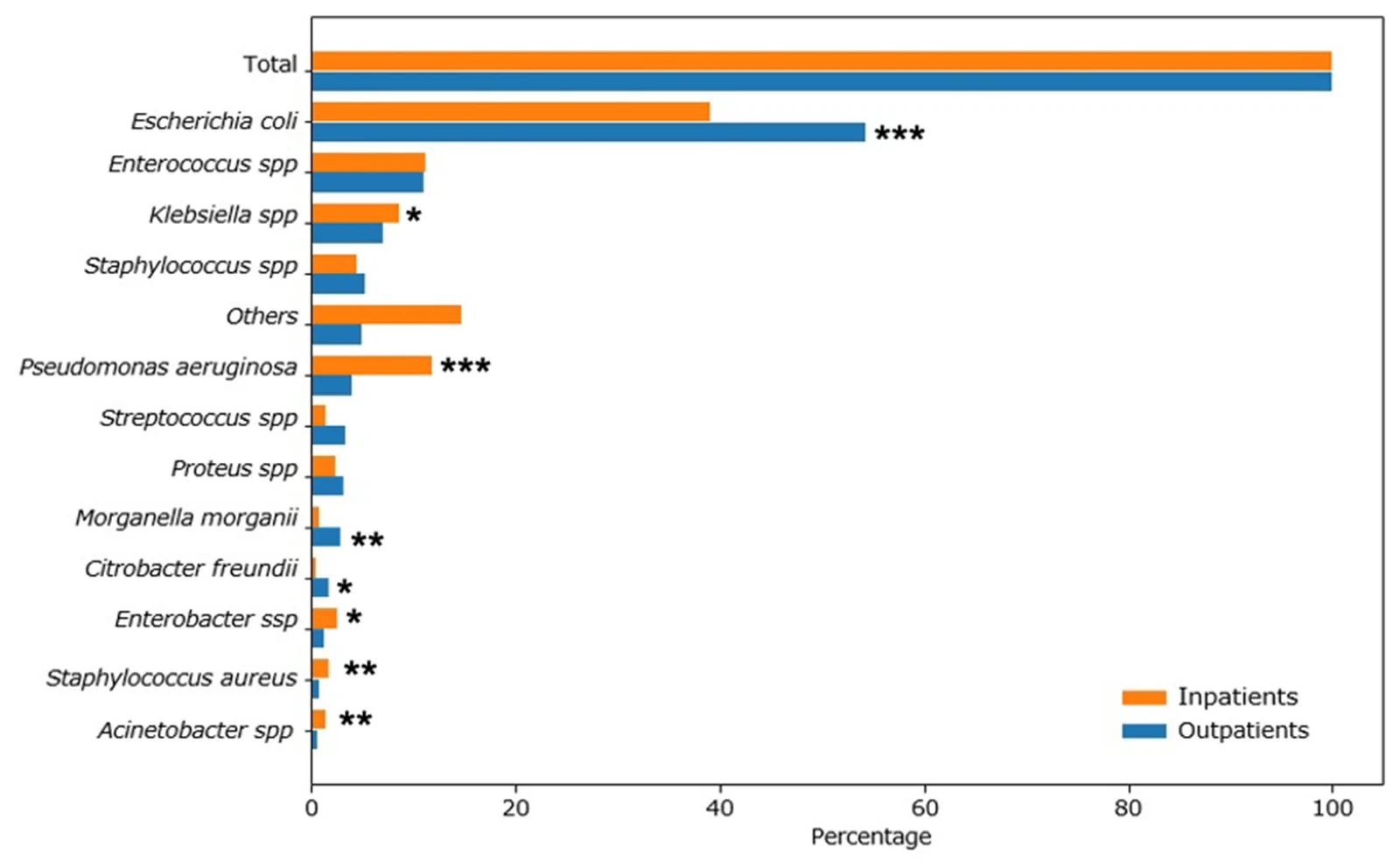
Representative bar plot of bacterial isolate frequency comparing microbial distributions between inpatients and outpatients from urine cultures. *p*-value < 0.05 was considered statistically significant (* *p* < 0.05, ** *p* < 0.01, *** *p* < 0.001).

We identified 3175 bacterial isolates obtained from urine samples, of which 79.9% were from outpatients and 20.1% from inpatients. *E. coli* was the predominant uropathogen in both groups, accounting for approximately 54.20% of the total cases. *E. coli* was significantly more frequently isolated in outpatients (*p* < 0.001). This was followed by *Enterococcus* spp., *Klebsiella* spp., *Enterobacter* spp., and *Proteus* spp. A higher percentage of *P. aeruginosa* was observed in inpatient isolates compared with outpatient isolates. Among inpatients, the probability of isolating this uropathogen was 3.69 times higher than among outpatients (OR 3.69; 95% IC: 2.74–4.95, *p* < 0.001) (Figure 1 and Table 2).

### 2.3. Antimicrobial Resistance Patterns of the Isolated Bacteria

**Figure 2:**
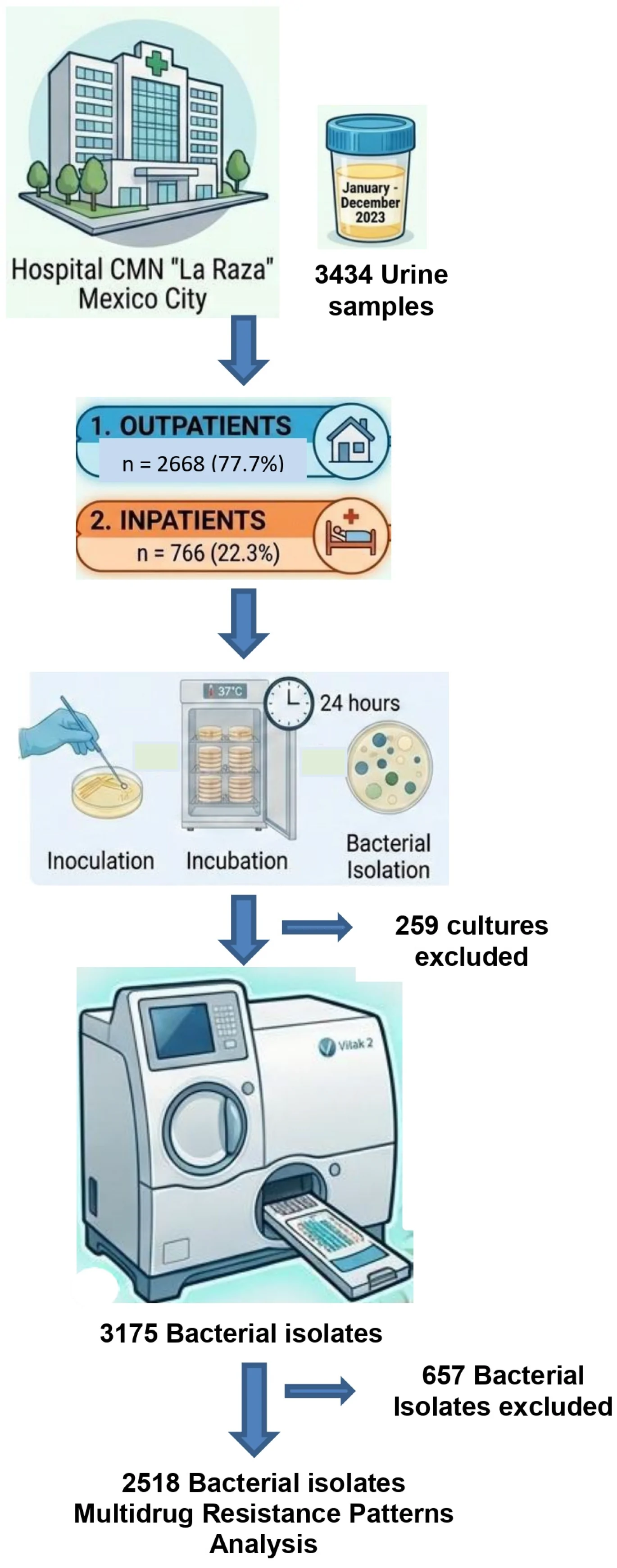
Flow diagram representing study design and number of samples at each step. Bacterial etiology and antimicrobial susceptibility are determined in outpatient and inpatient isolates at a tertiary care hospital in Mexico City.

AMR was observed in 3175 bacterial isolates obtained from urine samples (Figure 2). Among the outpatient isolates, *E. coli* showed carbapenem resistance. Meanwhile, in the inpatient isolates, resistance slightly increased. Resistance rates to third-generation cephalosporins and fluoroquinolones were higher in the inpatient isolates for CRO and CIP than in the outpatient isolates. *Klebsiella* spp. exhibited a similar pattern; inpatient isolates showed higher resistance to CXT, CRO, and GEN compared with outpatient isolates. Resistance to carbapenems was <9% in both groups, with a trend toward increasing resistance among inpatient isolates. *P. aeruginosa* showed lower resistance to meropenem (MRP) in outpatient isolates than in inpatient isolates. *Enterococcus* spp. showed resistance to ciprofloxacin (CIP) in inpatient isolates, higher than that in outpatient isolates. Meanwhile, *S. aureus* showed increased resistance to fluoroquinolones and β-lactams in inpatient isolates vs. outpatient isolates (Table 3).

### 2.4. Selected Multidrug-Resistance Patterns

In total, 2518 urinary bacterial isolates from patients were analyzed, and these isolates showed varying rates of selected MDR patterns (Figure 2). Among *E. coli* isolates, inpatient isolates showed higher resistance across nearly all tested antimicrobial combinations. Resistance rates to the combinations involving third-generation cephalosporins and fluoroquinolones (CRO, CIP, CF) reached 70.2% in inpatients compared to 48.4% in outpatients (OR 2.52; 95% IC: 1.92–3.29, *p* < 0.001). A substantial increase was noted for carbapenem-associated resistance combinations, such as ceftriaxone (CRO), CIP, and ertapenem (ETP). *E. coli* inpatient isolates showed more than a fivefold increase in MDR risk (OR 5.42; 95% IC: 3.05–9.63, *p* < 0.001).

*P. aeruginosa* showed significantly elevated resistance proportions among inpatients for combinations containing MRP (OR 3.14; 95% IC: 1.75–5.64, *p* < 0.001). *S. aureus* showed higher resistance in inpatient isolates vs. outpatient isolates for the combinations erythromycin (E) + clindamycin (CL) + oxacillin (OXI) and E + CL + levofloxacin (LEV). The results confirm that the highest rates of selected MDR patterns are observed in strains isolated from inpatient isolates, particularly *Enterobacter* spp., *Klebsiella* spp., *E. coli*, and *P. aeruginosa*, which were mainly resistant to combinations of third-generation cephalosporins, fluoroquinolones, and aminopenicillins (Table 4).

## 3. Discussion

AMR is a major global public health concern. By 2050, if insufficient action is taken for control, it may be responsible for 10 million annual deaths [1], placing a considerable financial burden on health systems worldwide [2]. Approximately 150 million people worldwide experience UTIs annually [3], and UTIs are the third leading cause of morbidity [10]. To identify the etiological agents and determine their AMR profiles in outpatient isolates and inpatient isolates at a tertiary-level hospital of the Mexican Social Security Institute in Mexico City, we analyzed 3175 urinary bacterial isolates.

The majority of the study population in both patient groups (outpatients and inpatients) consisted of women, which could be explained primarily by the anatomy of the female urinary tract (previously identified as a risk factor) [4]. Among inpatients, a higher proportion of males was observed compared with outpatients. Although our dataset did not capture the specific clinical interventions, the previous literature hypothesizes that this could be attributed to the use of invasive healthcare procedures and instruments [16]. The highest number of bacterial isolates was observed in the 18–54 age group in both patient groups. However, the proportion was higher among inpatients (50.3%) than among outpatients (44.6%). While not directly evaluated in our study, this plausibly reflects acute conditions or surgeries. Meanwhile, in the 65–74 age group, bacterial isolates were more frequent in outpatients (21.3%) than inpatients (17.2%), associated previously with home care and outpatient management of chronic infections. According to care services, 72.1% of outpatients came from the urology, nephrology, and kidney transplant services, whereas among inpatients, these services accounted for only 17.6%. These differences indicate a greater complexity in the hospitalized population and the involvement of hospital-based procedures (Table 1).

*E. coli* was the most frequently isolated pathogen in both patient groups. This finding is consistent with reports identifying this microorganism as the primary cause of community-acquired (75–95%) and hospital-acquired (50%) UTIs [17,18,19,20,21]. However, the frequencies reported here are lower than and contrast with some of the frequencies classically described [12]. We hypothesize that the lower proportion of *E. coli* in inpatient isolates may be attributed to a greater susceptibility to other opportunistic pathogens [6]. Regarding outpatients, a possible explanation is that they primarily come from specialized urology and nephrology clinics, with potentially more complicated or recurrent UTIs, where the etiology is more varied. The frequency of *P. aeruginosa* among inpatient isolates was higher than that among outpatient isolates, as was the case for *Acinetobacter* spp. While our database did not capture the duration of hospitalization or the specific use of medical devices, previous studies have consistently associated the presence of these bacteria with nosocomial infections [6,22], prolonged hospital stays, and catheterization [22,23].

In this study, *Klebsiella* spp. and *Enterobacter* spp. were more common among inpatient isolates than among outpatient isolates, whereas *Proteus* spp., *M. morganii*, and *C. freundii* predominated among outpatient isolates than among inpatient isolates. This is consistent with previous reports identifying *Klebsiella*, *Enterobacter*, *and Proteus* as common urinary pathogens, which are typically second only to *E. coli* in frequency among nosocomial infections [5,9,13].

The frequency of *Enterococcus* spp. was similar in both patient groups (*p* = 0.223), which falls within the range of traditionally reported frequencies in UTIs [5,8]. We observed a higher proportion of *S. aureus* in inpatient isolates than in outpatient isolates (2.03% vs. 0.71%) (*p* = 0.002). Both frequencies are below those reported internationally (1.0–5.3%) [5,24,25]. *Streptococcus* spp. was isolated more frequently from outpatient isolates than from inpatient isolates at a slightly higher rate than that reported in other studies (3.94%) [25]. In summary, these results indicate greater microbial heterogeneity in inpatient isolates, involving more Gram-positive bacteria (*Enterococcus*, *Staphylococcus*) than outpatient isolates in which Enterobacteriaceae predominate [7,8].

Our results revealed high rates of resistance to commonly used antimicrobials in inpatient isolates, for example, for *E. coli*, SXT, ampicillin (AMP), amoxicillin (AMX), and CIP. This is consistent with previous reports [21,25,26,27]. In contrast, we observed that *E. coli* and *K. pneumoniae* were generally less resistant (< 15.0%) to amikacin (AK) and MRP, which is consistent with the results of studies highlighting that these antimicrobials (AK, MRP) [18,19] and urinary-specific agents, such as FOS and nitrofurantoin (NIT), are generally the most effective against multidrug-resistant urinary pathogens [26]. In outpatients with UTIs, previous reports have suggested prioritizing MRP, NIT, and FOS because they are particularly active against *E. coli* and *Enterobacteriaceae* [21,26,28], and their use is supported when local resistance to quinolones or SXT is high [29]. In our case, the use of NIT and FOS would be justified against *E. coli*; however, the use of NIT against other bacteria of the *Enterobacteriaceae* family should be considered, as we observed resistance in some isolated strains. Regarding *S. aureus*, previous studies have reported sensitivity rates among strains isolated from UTIs to NIT and CIP [30]. In our study, we also observed high susceptibility to NIT but lower to CIP, with the lowest susceptibility observed in inpatient isolates. For SXT and fluoroquinolones, resistance rates of 40–50% have been reported [22,23], suggesting that these antimicrobials should be reserved or avoided unless susceptibility is confirmed. We observed susceptibility to SXT, but a wider and higher range of resistance to fluoroquinolones (LEV and MXF), with the highest resistance recorded in inpatient isolates.

Some reports recommend treating hospital-acquired UTIs caused by *P. aeruginosa* and *Acinetobacter* with CAZ based on the susceptibility profile [26]. However, we specifically observed CAZ resistance for *P. aeruginosa* and *Acinetobacter*. In summary, our results align with the global trends in AMR in urinary bacterial isolates, but regional particularities also exist.

The selected MDR patterns were observed in both outpatient and inpatient isolates, with generally higher frequencies among inpatient isolates. These observations are consistent with the global trend that nosocomial infections tend to involve more resistant pathogens in hospitals [31]. Because the present analysis was based on predefined antimicrobial combinations, these findings should be interpreted as specific selected MDR patterns rather than as overall MDR prevalence according to the consensus definition proposed by Magiorakos et al. [32].

*E. coli* exhibited the CRO–CIP–SAM MDR pattern in 51.0% of inpatient isolates compared with 31.0% of isolates in the outpatient community. This was lower than the 54.0% previously reported [33], suggesting the selection of more resistant strains in the hospital setting.

*Klebsiella* spp. exhibited the CRO–CIP–CF multidrug-resistance pattern in 52.0% of inpatient isolates compared with 33.0% of outpatient isolates. This pattern might suggest that inpatient isolates of *Klebsiella* and *E. coli* have increased their resistance to beta-lactams and quinolones (CIP), as extensively documented by the World Health Organization [17,31].

*S. aureus* exhibited the selected MDR patterns in 54.0% of the inpatient isolates (E, CL, OXI, and E, CL, LEV), compared with only 6.0% (E, CL, LEV) and 11.0% (E, CL, OXI) of the outpatient isolates. Inpatient isolates of *P. aeruginosa* showed selected MDR pattern rates exceeding 63.0% (CAZ, CIP, AK) compared with 40.0% among outpatient isolates, which could indicate the selection of MDR strains in hospital settings.

Our results showed selected MDR patterns in urinary bacterial isolates, especially among inpatient isolates. This is consistent with global trends of increasing MDR [31], making it imperative to strengthen surveillance measures and implement policies for the rational use of antimicrobials, such as promoting prescriptions based on culture results, susceptibility testing, and periodic reviews of local antibiotic use protocols, as recommended by expert sources [31]. Such actions can optimize therapeutic outcomes, minimize complications, and slow the progression of drug resistance.

### Limitations

This study has some limitations. First, it corresponds to a retrospective design. A single-center design at a tertiary referral hospital may limit the generalizability of the findings and introduce referral bias, with an overrepresentation of patients from specialized urology, nephrology, and kidney transplantation services. Second, antimicrobial resistance was assessed only by phenotypic susceptibility testing. No data are available on the specific mechanisms of resistance to ESBLs, carbapenemases, methicillin, vancomycin, aminoglycosides, ceftriaxone, and carbapenems. Molecular characterization of resistance mechanisms, including ESBLs, carbapenemases, and methicillin resistance, was not performed. Third, the retrospective laboratory-based database lacked relevant patient-level clinical information such as urinary symptoms, systemic signs, pyuria, clinician diagnosis, antimicrobial treatment, pregnancy, urinary catheterization, urinary tract abnormalities, or other clinical criteria. Therefore, symptomatic urinary tract infection could not be distinguished from asymptomatic bacteriuria, and independent clinical risk factors for antimicrobial resistance and multidrug resistance could not be identified. Fourth, the use of a universal threshold of 1.0 × 10^5^ CFU/mL may exclude clinically significant infections, particularly in symptomatic patients. Fifth, infections could not be classified as community-acquired, healthcare-associated, or hospital-acquired according to standardized criteria. Therefore, comparisons between outpatients and inpatients should be interpreted as differences between clinical settings rather than infection origin. Finally, several organisms were analyzed at the genus level, limiting species-specific comparisons. The relatively small number of isolates for some pathogens reduced the precision of resistance estimates (*C. freundii*). Clinical outcomes, including treatment response, recurrence, length of hospital stay, and mortality, were unavailable. Therefore, the clinical impact of antimicrobial resistance could not be evaluated. Furthermore, since the selected MDR patterns were assessed through numerous exploratory comparisons without multiplicity adjustment, these findings should be interpreted with caution. Prospective multicenter studies integrating clinical, microbiological, molecular, and epidemiological data are needed to validate and extend these findings.

## 4. Materials and Methods

### 4.1. Study Design, Population, Sample Size, and Ethical Aspects

In this retrospective, cross-sectional, laboratory-based surveillance study, we analyzed a census of all eligible urine cultures processed from 1 January to 31 December 2023 at the “Dr. Antonio Fraga Mouret” Hospital de Especialidades, Centro Médico Nacional (CMN) “La Raza”, IMSS in Mexico City, Mexico. Adult patients (≥18 years) receiving care as either outpatients or inpatients were eligible for inclusion (Figure 2). For patients with more than one urine culture during the study period, only the first eligible isolate per patient was retained to avoid overrepresentation of recurrent cultures in the epidemiological analysis. Samples with negative urine cultures, contaminated specimens, and mixed bacterial growth considered noninterpretable were excluded from the antimicrobial resistance analysis. After applying these exclusion criteria, 3175 bacterial isolates with valid antimicrobial susceptibility results were included in the antimicrobial resistance analysis. Only one isolate per patient was considered. The laboratory procedures were performed in the Medical Bacteriology section of the Central Clinical Analysis Laboratory at the same hospital. Sociodemographic data (age, sex, and medical services) were collected using a test request form submitted by the treating physician. This study was approved by the Research and Ethics Committee of Dr. Antonio Fraga Mouret Hospital de Especialidades CMN “La Raza”, IMSS, Mexico City, Mexico (authorization number: R-2025-3501-288) in November 2025. Our research involved the collection of data from medical records and the handling of biological samples, that is, the collection and analysis of which posed no risk to patients. We requested an exemption from the informed consent form from the same committee, and our request was approved in July 2025. Participant identification numbers were used in the study instead of names to ensure confidentiality.

### 4.2. Sample Collection

Outpatient study participants collected their urine samples according to the IMSS guidelines, whereas nursing staff assisted the hospitalized participants in collecting their urine. They collected 10–20 mL of urine in a sterile, wide-mouth plastic container with a screw cap. The staff promptly labeled each sample with the patient’s information and processed it within 2 h.

### 4.3. Urine Culture

We performed quantitative urine culture to detect significant bacteriuria. We inoculated the samples onto CHROMagar^®^ Orientation, MacConkey Agar, and Blood Agar plates (Becton, Dickinson and Company, Sparks, MD, USA) using a calibrated loop of approximately 10 µL. The plates were then incubated under aerobic conditions at 37 °C for 24 h. We defined significant bacteriuria as a urine colony count ≥ 1.0 × 10^5^ colony-forming units (CFU)/mL. Samples with values < 1.0 × 10^5^ CFU/mL were classified as insignificant or suggestive of contamination. For polymicrobial growth, isolation of more than two microbial isolates from a urine culture is associated with contamination. For two microbial isolates from a urine culture, sub-cultures were performed to obtain individual isolates from each organism. However, only the microorganism with the highest CFU/mL from the primary culture was included in the identification and antimicrobial susceptibility testing.

### 4.4. Definition of Terms

Cultures were quantitated, and significant bacteriuria was defined as a specimen containing bacterial species ≥ 1.0 × 10^5^ CFU/mL of urine according to the standard guideline [34].

Multidrug resistance (MDR) was defined as resistance to at least one antimicrobial agent in three or more antimicrobial families [32].

### 4.5. Bacterial Identification and Antimicrobial Susceptibility

Bacterial identification and antimicrobial susceptibility testing were performed using the VITEK 2 XL automated system (bioMérieux^®^, Marcy-l’Étoile, France). According to the manufacturer’s instructions, VITEK^®^ 2 ID-GNB cards identified Gram-negative bacilli, while VITEK^®^ 2 GP cards identified Gram-positive bacteria. Susceptibility testing used VITEK^®^ 2 AST-N401 cards for Gram-negative bacilli and AST-P663 cards for Gram-positive bacteria. Results were interpreted using the software version VTK-9.02, an advanced expert system (AES). AES is based on >2000 phenotypes and 20,000 MIC distributions obtained from the published literature, internal bioMérieux data, and external experts. After identification and susceptibility testing, the AES searches its knowledge base for common MIC distributions to check for consistency with established phenotypes, thus establishing biological validation. This comparison allows AES to determine whether identification matches the susceptibility pattern and whether MICs fit a specific phenotype. At the end of each cycle, a printout of the MIC values of the antibiotics was obtained and recorded.

### 4.6. Quality Control

Quality control procedures for VITEK 2 XL identification and MIC-based susceptibility testing were performed according to CLSI and bioMérieux guidelines. Daily QC included *E. coli* (ATCC 25922) and *S. aureus* (ATCC 25923). Additional reference strains were tested weekly and with each new lot of AST cards: *K. pneumoniae* (ATCC 700603), *P. aeruginosa* (ATCC 27853), *A. baumannii* (ATCC 19606), and *E. faecalis* (ATCC 29212). Instrument calibration was verified annually.

### 4.7. Statistical Analysis and Data Preparation

Categorical variables were summarized as frequencies and percentages. Differences in sociodemographic characteristics, namely, age groups, sex, and medical service of origin, between outpatients and inpatients were evaluated using Pearson’s Chi-square test (χ^2^). A two-sided *p*-value < 0.05 was considered statistically significant.

Categorical variables representing bacterial isolation frequencies were summarized as absolute counts (*n*) and relative percentages (%). Differences in the distribution of uropathogens between outpatients and inpatients were evaluated using Pearson’s Chi-square (χ^2^) test of independence on a 12 × 2 contingency matrix. To assess the specific association of individual bacterial species with patient setting (inpatients vs. outpatients), two-by-two (2 × 2) contingency tables were analyzed to calculate Odds Ratios (OR) with corresponding 95% Confidence Intervals (CI), using the outpatient group as a reference. To control for Type I error arising from multiple hypothesis testing across 12 bacterial groups, a Bonferroni correction was applied, setting the threshold for statistical significance at *p* < 0.0042 (α = 0.05/12).

The number of each isolated bacterial species was expressed as absolute frequencies (*n*) and relative percentages (%) relative to the total number of bacterial isolates recovered from patient urine samples. Antimicrobial resistance profiles were calculated per bacterial species using descriptive statistics.

Categorical variables were expressed as absolute and relative frequencies. To assess the association and compare multidrug-resistance profiles between bacterial isolates from outpatients and inpatients, Fisher’s exact test and Pearson’s chi-square test (χ^2^) were used as appropriate. The odds ratio (OR) and its respective confidence intervals were calculated to estimate the magnitude of the risk of resistance in inpatient isolates. Documented intrinsic resistance (IR) was excluded from the corresponding comparative analysis. A statistical significance level of *p* < 0.05 was considered. Given the exploratory nature of the selected MDR pattern comparisons across various bacterial species and antimicrobial combinations, no formal multiplicity adjustment was applied to these specific analyses to retain sensitivity for detecting potential resistance signals. Statistical analyses were performed using Python (SciPy library, v1.11).

### 4.8. Descriptive Analysis of the Isolates

The descriptive analysis included the 3175 eligible bacterial isolates recovered from urine cultures with significant bacteriuria after applying the predefined exclusion criteria. Subsequently, the frequency and percentage of isolated bacteria, including *E. coli*, *Enterococcus* spp., *Klebsiella* spp., *P. aeruginosa*, *Proteus* spp., *M. morganii*, *C. freundii*, *Enterobacter* spp., *Acinetobacter* spp., *Staphylococcus* spp., *S. aureus*, and *Streptococcus* spp., were analyzed in outpatient and inpatient settings to determine the prevalence of these microorganisms.

### 4.9. Analysis of Susceptibility Profiles

Antimicrobial susceptibility profiles were evaluated using MIC values generated by the VITEK^®^ according to the Clinical and Laboratory Standards Institute (CLSI) interpretative criteria in force during the study period. Isolates were classified as susceptible (S), intermediate (I), or resistant (R) for each antimicrobial agent. The corresponding percentages were calculated separately for the outpatient and inpatient isolates [35]. Isolates categorized as intermediate or susceptible were excluded from the resistance calculations.

### 4.10. Analysis of Selected Multidrug-Resistance Patterns

Selected MDR pattern analysis was performed only on bacterial isolates with complete and valid antimicrobial susceptibility results that allowed the evaluation of resistance across at least three antimicrobial classes. Of the 3175 bacterial isolates included in the antimicrobial resistance analysis, 2518 fulfilled these criteria and were, therefore, eligible for selected multidrug resistance pattern analysis. Isolates with incomplete susceptibility profiles or insufficient antimicrobial testing to determine selected MDR pattern status were excluded from this analysis.

To characterize selected MDR patterns, predefined combinations of antimicrobials from three different families were evaluated. For each combination, the percentage of bacterial isolates showing simultaneous resistance to all antimicrobial agents included in the combination was calculated (Table 4). These predefined combinations were analyzed as specific resistance patterns and were not used to estimate overall MDR prevalence according to the consensus definition proposed by Magiorakos et al. [32].

## 5. Conclusions

The etiological distribution and in vitro antimicrobial resistance of urinary bacterial isolates differ between outpatient and inpatient isolates. Although *Escherichia coli* is the predominant microorganism in both groups, inpatient isolates demonstrate a higher frequency of opportunistic and nosocomial pathogens, including *Pseudomonas aeruginosa*, *Klebsiella* spp., *Enterobacter* spp., and *Staphylococcus aureus*. These findings suggest that our results, while indicating local nuances, are consistent with global trends. These preliminary insights could help inform regional stewardship strategies and guide initial antibiotic choices in our hospital.

## Figures and Tables

**Table 1:** Sociodemographic characteristics of patients treated at the Hospital de Especialidades, CMN “La Raza”, IMSS, in Mexico City between January and December 2023.

Sociodemographic Characteristic	Outpatients (*n* = 2668)	Inpatients (*n* = 766)	Total (N = 3434)	*p*-Value
Age group (years)				0.022 *
18–54	1191 (44.6)	385 (50.3)	1576 (45.9)	
55–64	604 (22.6)	170 (22.2)	774 (22.5)	
65–74	567 (21.3)	132 (17.2)	699 (20.4)	
>75	306 (11.5)	79 (10.3)	385 (11.2)	
Sex				0.012 *
Male	1034 (38.8)	336 (43.9)	1370 (39.9)	
Female	1634 (61.2)	430 (56.1)	2064 (60.1)	
Medical service				<0.001 *
Internal Medicine	54 (2.0)	146 (19.1)	200 (5.8)	
Urology/Nephrology/Kidney Transplant	1924 (72.1)	135 (17.6)	2059 (60.0)	
Neurology/Neurosurgery	38 (1.4)	111 (14.5)	149 (4.3)	
Intensive Care Unit (ICU)	10 (0.4)	89 (11.6)	99 (2.9)	
Cardiology/Angiology	15 (0.6)	72 (9.4)	87 (2.5)	
Others	627 (23.5)	213 (27.8)	840 (24.5)	

Note: A total of 3434 urine samples from outpatients and inpatients, from several medical services, between 18 and 75 years old, both men and women, were analyzed. Data are presented as absolute frequencies (*n*) and percentages (%). * Calculated using Pearson’s Chi-square test (χ^2^); ICU (Intensive Care Unit).

**Table 2:** Frequency of bacteria isolated from urine cultures of patients treated at the Hospital de Especialidades CMN “La Raza”, IMSS, in Mexico City between January and December 2023.

Bacterial Isolate	Outpatients (N = 2536) *n* (%)	Inpatients (N = 639) *n* (%)	Total Isolates (N = 3175) *n* (%)	Odds Ratio (95% CI) *	*p*-Value †
*Escherichia coli*	1432 (56.5%)	289 (45.2%)	1721 (54.2%)	0.64 (0.53–0.76)	<0.001
*Enterococcus* spp.	293 (11.6%)	85 (13.3%)	378 (11.9%)	1.17 (0.91–1.52)	0.223
*Klebsiella* spp.	186 (7.3%)	66 (10.3%)	252 (7.9%)	1.46 (1.08–1.96)	0.012
*Pseudomonas aeruginosa*	108 (4.3%)	90 (14.1%)	198 (6.2%)	3.69 (2.74–4.95)	<0.001
*Proteus* spp.	83 (3.3%)	18 (2.8%)	101 (3.2%)	0.86 (0.51–1.44)	0.557
*Morganella morganii*	79 (3.1%)	6 (0.9%)	85 (2.7%)	0.29 (0.13–0.68)	0.002
*Citrobacter freundii*	46 (1.8%)	3 (0.5%)	49 (1.5%)	0.26 (0.08–0.82)	0.014
*Enterobacter* spp.	35 (1.4%)	19 (3.0%)	54 (1.7%)	2.19 (1.24–3.85)	0.005
*Acinetobacter* spp.	17 (0.7%)	10 (1.6%)	27 (0.9%)	2.36 (1.07–5.17)	0.028
*Staphylococcus* spp.	139 (5.5%)	34 (5.3%)	173 (5.4%)	0.97 (0.66–1.42)	0.873
*Staphylococcus aureus*	18 (0.7%)	13 (2.0%)	31 (1.0%)	2.91 (1.42–5.96)	0.002
*Streptococcus* spp.	100 (3.9%)	6 (0.9%)	106 (3.3%)	0.23 (0.10–0.53)	<0.001

Note: Comparison of percentages and statistical results of bacterial isolates between the study groups. Percentages are calculated based on the total number of isolates within each patient group. * Odds Ratio (OR) with 95% Confidence Interval (CI) calculated for Inpatients relative to Outpatients. † *p*-values derived from Pearson’s Chi-square test (or Fisher’s exact test where appropriate) for individual 2 × 2 contingency tables. *p*-values indicate statistical significance after Bonferroni correction for multiple comparisons (threshold α = 0.05/12 = 0.0042).

**Table 3:** Antimicrobial resistance rates of bacteria isolates from patients treated at the Hospital de Especialidades CMN “La Raza”, IMSS, in Mexico City between January and December 2023.

Bacteria Isolates	*n*	%	GEN	AK	ETP	MRP	DOR	CF	CXM	CXMA	CXT	CRO
Outpatients	2536	100	*n* (%)	*n* (%)	*n* (%)	*n* (%)	*n* (%)	*n* (%)	*n* (%)	*n* (%)	*n* (%)	*n* (%)
*Escherichia coli*	1432	56.5	376 (26.25)	165 (11.52)	27 (1.88)	15 (1.04)	0 (0)	832 (58.10)	179 (12.5)	179 (12.5)	707 (49.37)	773 (53.98)
*Klebsiella* spp.	186	7.3	50 (26.88)	6 (3.23)	5 (2.69)	1 (0.54)	0 (0)	77 (41.40)	9 (4.84)	9 (4.84)	65 (34.95)	76 (40.86)
*P. aeruginosa*	108	4.3	11 (10.19)	51 (47.22)	N/A	42 (38.88)	0 (0)	N/A	N/A	N/A	19 (17.59)	N/A
*Proteus* spp.	83	3.3	27 (32.53)	1 (1.20)	0 (0)	0 (0)	N/A	24 (28.92)	5 (6.02)	5 (6.02)	17 (20.48)	16 (19.28)
*Morganella morganii*	79	3.1	24 (30.38)	1 (1.27)	1 (1.27)	0 (0)	N/A	16 (20.25)	16 (20.2)	16 (20.25)	7 (8.86)	4 (5.06)
*Citrobacter freunaii*	46	1.8	11 (23.91)	5 (10.87)	6 (13.04)	2 (4.35)	N/A	9 (19.57)	9 (19.57)	9 (19.57)	15 (31.61)	17 (36.96)
*Enterobacter* spp.	35	1.4	5 (14.29)	5 (14.29)	5 (14.29)	2 (5.71)	N/A	5 (14.29)	5 (14.29)	5 (14.29)	11 (31.43)	11 (31.43)
*Acinetobacter* spp.	17	0.7	3 (17.65)	0 (0)	N/A	3 (17.65)	N/A	6 (35.29)	1 (5.88)	N/A	3 (17.65)	3 (17.65)
			DAP	DO	E	MRP	LEV	TGC	LNZ	VA	CXT	CRO
*Enterococcus* spp.	293	11.6	0 (0)	83 (28.32)	135 (46.07)	2 (0.68)	94 (32.08)	0 (0)	14 (4.77)	13 (4.43)	N/A	N/A
			GEN	RA	OXI	MRP	CAZ	CIP	LEV	MXF	SXT	VA
*Staphylococcus* spp.	139	5.5	21 (15.11)	5 (3.60)	99 (71.22)	2 (1.44)	18 (12.95)	61 (43.88)	69 (49.64)	40 (28.78)	44 (31.65)	2 (1.44)
*S. aureus*	18	0.7	0 (0)	0 (0)	5 (27.78)	0 (0)	3 (16.67)	2 (11.11)	2 (11.11)	1 (5.56)	0 (0)	0 (0)
*Streptococcus* spp.	100	3.9	N/A	N/A	N/A	0 (0)	10 (10.0)	N/A	5 (5.0)	5 (5.0)	N/A	0 (0)
**Bacteria Isolates**	***n***	**%**	**CAZ**	**FEF**	**NOR**	**CIP**	**SXT**	**NIT**	**AMP**	**AMX**	**SAM**	**FOS**
**Outpatients**	**2536**	**100**	***n* (%)**	***n* (%)**	***n* (%)**	***n* (%)**	***n* (%)**	***n* (%)**	***n* (%)**	***n* (%)**	***n* (%)**	***n* (%)**
*Escherichia coli*	1432	56.5	179 (12.50)	764 (53.35)	987 (68.92)	771 (53.84)	789 (55.10)	115 (8.03)	254 (17.74)	755 (52.72)	617 (43.09)	63 (4.40)
*Klebsiella* spp.	186	7.3	75 (40.32)	75 (40.32)	58 (31.18)	81 (43.55)	85 (45.70)	61 (32.80)	20 (10.75)	161 (86.56)	15 (8.06)	9 (4.84)
*P. aeruginosa*	108	4.3	43 (39.81)	42 (38.88)	46 (42.59)	51 (47.22)	N/A	N/A	N/A	50 (46.29)	7 (6.48)	N/A
*Proteus* spp.	83	3.3	9 (10.84)	16 (19.28)	9 (10.84)	36 (43.37)	44 (53.01)	19 (22.89)	12 (14.46)	31 (37.35)	8 (9.64)	4 (4.82)
*Morganella morganii*	79	3.1	9 (11.39)	2 (2.53)	9 (11.39)	62 (78.48)	46 (58.23)	16 (20.25)	16 (20.25)	68 (86.08)	4 (5.06)	13 (16.46)
*Citrobacter freunaii*	46	1.8	16 (34.78)	9 (19.57)	22 (47.83)	32 (69.57)	23 (50.00)	7 (15.22)	N/A	37 (80.43)	7 (15.22)	0 (0)
*Enterobacter* spp.	35	1.4	8 (22.86)	10 (28.57)	9 (25.71)	11 (31.43)	9 (25.71)	9 (25.71)	N/A	33 (94.29)	2 (5.71)	4 (11.43)
*Acinetobacter* spp.	17	0.7	3 (17.65)	3 (17.65)	N/A	3 (17.65)	0 (0)	N/A	N/A	7 (41.18)	4 (23.53)	N/A
			CAZ	FEF	NOR	CIP	SXT	NIT	AMP	AMX	SAM	FOS
*Enterococcus* spp.	293	11.6	32 (10.92)	N/A	N/A	96 (32.76)	N/A	14 (4.78)	18 (6.14)	23 (7.85)	N/A	N/A
			TGC	CL	DAP	E	NIT	LNZ	AMP	SAM	DO	TE
*Staphylococcus* spp.	139	5.5	0 (0)	71 (51.08)	0 (0)	106 (76.26)	1 (0.72)	2 (1.44)	N/A	13 (9.35)	10 (7.19)	35 (25.18)
*S. aureus*	18	0.7	0 (0)	5 (27.78)	0 (0)	8 (44.44)	0 (0)	0 (0)	N/A	3 (16.67)	0 (0)	2 (11.11)
*Streptococcus* spp.	100	3.9	0 (0)	N/A	N/A	N/A	3 (3.0)	0 (0)	0 (0)	4 (4.0)	N/A	47 (47.0)
**Bacteria Isolates**	***n***	**%**	**GEN**	**AK**	**ETP**	**MRP**	**DOR**	**CF**	**CXM**	**CXMA**	**CXT**	**CRO**
**Inpatients**	**639**	**100**	***n* (%)**	***n* (%)**	***n* (%)**	***n* (%)**	***n* (%)**	***n* (%)**	***n* (%)**	***n* (%)**	***n* (%)**	***n* (%)**
*Escherichia coli*	289	45.2	88 (30.45)	42 (14.53)	28 (9.69)	15 (5.19)	0 (0)	227 (78.55)	54 (18.69)	54 (18.69)	209 (72.32)	224 (77.51)
*Klebsiella* spp.	66	10.3	25 (37.88)	6 (9.09)	6 (9.09)	5 (7.57)	N/A	43 (65.15)	6 (9.09)	6 (9.09)	39 (59.09)	45 (68.18)
*P. aeruginosa*	90	14.1	15 (16.67)	57 (63.33)	0 (0)	60 (66.66)	N/A	N/A	N/A	N/A	20 (22.22)	0 (0)
*Proteus* spp.	18	2.8	6 (33.33)	0 (0)	0 (0)	0 (0)	N/A	5 (27.78)	1 (5.56)	1 (5.56)	3 (16.67)	4 (22.22)
*Morganella morganii*	6	0.9	1 (16.67)	0 (0)	0 (0)	0 (0)	N/A	1 (16.67)	1 (16.67)	1 (16.67)	0 (0)	0 (0)
*Citrobacter freunaii*	3	0.5	1 (33.33)	1 (33.33)	1 (33.33)	1 (33.33)	N/A	N/A	N/A	N/A	1 (33.33)	1 (33.33)
*Enterobacter* spp.	19	3.0	3 (15.79)	14 (73.68)	5 (26.32)	1 (5.26)	N/A	2 (10.53)	2 (10.53)	2 (10.53)	15 (78.95)	17 (89.47)
*Acinetobacter* spp.	10	1.6	6 (60.0)	1 (10.0)	N/A	8 (80.0)	N/A	N/A	N/A	N/A	8 (80.0)	9 (90.0)
			DAP	DO	E	MRP	LEV	TGC	LNZ	VA	CXT	CRO
*Enterococcus* spp.	85	13.3	0 (0)	15 (17.64)	57 (67.05)	2 (2.35)	48 (56.47)	0 (0)	1 (1.17)	18 (21.17)	N/A	N/A
			GEN	RA	OXI	MRP	CAZ	CIP	LEV	MXF	SXT	VA
*Staphylococcus* spp.	34	5.3	10 (29.41)	5 (14.71)	33 (97.06)	0 (0)	4 (11.76)	16 (47.06)	21 (61.76)	15 (44.12)	8 (23.53)	1 (2.94)
*S. aureus*	13	2.0	0 (0)	0 (0)	7 (53.85)	0 (0)	0 (0)	8 (61.54)	8 (61.54)	8 (61.54)	0 (0)	0 (0)
*Streptococcus* spp.	6	0.9	N/A	N/A	N/A	0 (0)	1 (16.67)	N/A	0 (0)	0 (0)	N/A	0 (0)
**Bacteria Isolates**	***n***	**%**	**CAZ**	**FEF**	**NOR**	**CIP**	**SXT**	**NIT**	**AMP**	**AMX**	**SAM**	**FOS**
**Inpatients**	**639**	**100**	***n* (%)**	***n* (%)**	***n* (%)**	***n* (%)**	***n* (%)**	***n* (%)**	***n* (%)**	***n* (%)**	***n* (%)**	***n* (%)**
*Escherichia coli*	289	45.2	225 (77.85)	216 (74.74)	225 (77.85)	250 (86.51)	193 (66.78)	13 (4.50)	62 (21.45)	194 (67.13)	175 (60.55)	12 (4.15)
*Klebsiella* spp.	66	10.3	44 (66.67)	43 (65.15)	19 (28.79)	39 (59.09)	42 (63.64)	25 (37.88)	8 (12.12)	58 (87.88)	37 (56.06)	1 (1.52)
*P. aeruginosa*	90	14.1	62 (68.88)	60 (66.67)	61 (67.78)	63 (70.00)	N/A	N/A	N/A	82 (91.11)	13 (14.44)	0 (0)
*Proteus* spp.	18	2.8	4 (22.22)	3 (16.67)	2 (11.11)	10 (55.56)	10 (55.56)	3 (16.67)	1 (5.56)	4 (22.22)	2 (11.11)	1 (5.56)
*Morganella morganii*	6	0.9	0 (0)	0 (0)	0 (0)	5 (83.33)	3 (50.0)	1 (16.67)	1 (16.67)	3 (50.0)	3 (50.0)	1 (16.67)
*Citrobacter freunaii*	3	0.5	1 (33.33)	1 (33.33)	1 (33.33)	2 (66.67)	2 (66.67)	1 (33.33)	N/A	3 (100.0)	N/A	N/A
*Enterobacter* spp.	19	3.0	16 (84.21)	16 (84.21)	15 (78.95)	16 (84.21)	17 (89.47)	14 (73.68)	N/A	16 (84.21)	1 (5.26)	2 (10.53)
*Acinetobacter* spp.	10	1.6	9 (90.0)	8 (80.0)	N/A	9 (90.0)	2 (20.0)	N/A	N/A	8 (80.0)	7 (70.0)	N/A
			CAZ	FEF	NOR	CIP	SXT	NIT	AMP	AMX	SAM	FOS
*Enterococcus* spp.	85	13.3	N/A	N/A	N/A	48 (56.47)	N/A	17 (20.00)	26 (30.59)	10 (11.76)	N/A	N/A
			TGC	CL	DAP	E	NIT	LNZ	AMP	SAM	DO	TE
*Staphylococcus* spp.	34	5.3	0 (0)	23 (67.65)	0 (0)	28 (82.35)	0 (0)	0 (0)	N/A	3 (8.82)	5 (14.71)	12 (35.29)
*S. aureus*	13	2.0	0 (0)	8 (61.54)	0 (0)	8 (61.54)	0 (0)	0 (0)	N/A	2 (15.38)	0 (0)	0 (0)
*Streptococcus* spp.	6	0.9	0 (0)	N/A	N/A	N/A	0 (0)	0 (0)	0 (0)	0 (0)	N/A	3 (50.0)

Key terms: AMP, ampicillin; AMX, amoxicillin; AK, amikacin; CAZ, ceftazidime; CF, cefalotin; CIP, ciprofloxacin; CL, clindamycin; CRO, ceftriaxone; CXT, cefotaxime; CXMA, cefuroxime axetil; CXM, cefuroxime; DAP, daptomycin; DO, doxycycline; DOR, doripenem; E, erythromycin; ETP, ertapenem; FEF, cefepime; FOS, fosfomycin; NIT, nitrofurantoin; GEN, gentamicin; LEV, levofloxacin; LNZ, linezolid; MRP, meropenem; MXF, moxifloxacin; NOR, norfloxacin; OXI, oxacillin; RA, rifampicin; SAM, ampicillin/sulbactam; SXT, trimethoprim/sulfamethoxazole; TGC, tigecycline; TE, tetracycline; VA, vancomycin; N/A, not applicable. Data are presented as absolute frequencies (*n*) and relative percentages (*%*) of the total bacterial isolates recovered from patient urine samples. Antimicrobial resistance profiles were determined per bacterial species using descriptive statistics.

**Table 4:** Selected multidrug-resistance patterns among bacterial isolates from patients treated at the Hospital de Especialidades, CMN “La Raza”, IMSS in Mexico City between January and December 2023.

Bacterial Isolate	Combination Antimicrobial	Outpatients (*n*/N)	Outpatients % MDR	Inpatients (*n*/N)	Inpatients % MDR	Odds Ratio	*p*-Value
*E. coli* (N = 1748)							
	CRO, CIP, SAM	447/1449	0.31	151/299	0.51	2.29 (1.78—2.94)	*p* < 0.001 ***
	CRO, CIP, CF	701/1449	0.48	210/299	0.70	2.52 (1.92—3.29)	*p* < 0.001 ***
	CRO, CIP, SXT	416/1449	0.29	138/299	0.46	2.13 (1.65—2.74)	*p* < 0.001 ***
	CRO, CIP, AMX	619/1449	0.43	175/299	0.59	1.89 (1.47—2.44)	*p* < 0.001 ***
	CRO, CIP, GEN	257/1449	0.18	71/299	0.24	1.44 (1.07—1.95)	*p* < 0.05 *
	CRO, CIP, ETP	24/1449	0.02	25/299	0.08	5.42 (3.05—9.63)	*p* < 0.001 ***
	CRO, CIP, FOS	47/1449	0.03	12/299	0.04	1.25 (0.65—2.38)	NS
*Enterobacter* spp. (N = 54)							
	CRO, CIP, SXT	6/35	0.17	16/19	0.84	25.78 (5.67—117.20)	*p* < 0.001 ***
	CRO, CIP, GEN	4/35	0.11	3/19	0.16	1.45 (0.29—7.30)	NS
	CRO, CIP, ETP	5/35	0.14	5/19	0.26	2.14 (0.53—8.62)	NS
	CRO, CIP, FOS	0/35	0.00	2/19	0.11	N/A	NS
*Klebsiella* spp. (N = 252)							
	CRO, CIP, CF	61/186	0.33	34/66	0.52	2.18 (1.23—3.86)	*p* < 0.01 **
	CRO, CIP, SXT	56/186	0.30	32/66	0.48	2.18 (1.23—3.88)	*p* < 0.05 *
	CRO, CIP, GEN	42/186	0.23	23/66	0.35	1.83 (0.99—3.38)	NS
	CRO, CIP, ETP	4/186	0.02	4/66	0.06	2.94 (0.71—12.09)	NS
	CRO, CIP, FOS	1/186	0.01	1/66	0.02	2.85 (0.18—46.16)	NS
*Proteus* spp. (N = 101)							
	CRO, CIP, SAM	9/83	0.11	0/18	0.00	N/A	NS
	CRO, CIP, SXT	13/83	0.16	3/18	0.17	1.08 (0.27—4.25)	NS
	CRO, CIP, GEN	10/83	0.12	2/18	0.11	0.91 (0.18—4.57)	NS
	CRO, CIP, ETP	0/83	0.00	0/18	0.00	N/A	NS
	CRO, CIP, FOS	1/83	0.01	1/18	0.06	4.82 (0.29—80.98)	NS
*M. morganii* (N = 85)							
	CRO, CIP, SAM	3/79	0.04	0/6	0.00	N/A	NS
	CRO, CIP, SXT	4/79	0.05	0/6	0.00	N/A	NS
	CRO, CIP, GEN	0/79	0.00	0/6	0.00	N/A	NS
	CRO, CIP, ETP	0/79	0.00	0/6	0.00	N/A	NS
	CRO, CIP, FOS	2/79	0.03	0/6	0.00	N/A	NS
*C. freundii* (N = 49)							
	CRO, CIP, SXT	12/46	0.26	1/3	0.33	1.42 (0.12—17.07)	NS
	CRO, CIP, AMX	15/46	0.33	1/3	0.33	1.03 (0.09—12.32)	NS
	CRO, CIP, GEN	9/46	0.20	1/3	0.33	2.06 (0.17—25.26)	NS
	CRO, CIP, ETP	6/46	0.13	1/3	0.33	3.33 (0.26—42.66)	NS
	CRO, CIP, FOS	0/46	0.00	0/3	0.00	N/A	NS
*P. aeruginosa* (N = 198)							
	CAZ, CIP, AK	43/108	0.40	57/90	0.63	2.61 (1.47—4.65)	*p* < 0.01 **
	CAZ, CIP, MRP	42/108	0.39	60/90	0.67	3.14 (1.75—5.64)	*p* < 0.001 ***
*S. aureus* (N = 31)							
	E, CL, OXI	2/18	0.11	7/13	0.54	9.33 (1.50—58.20)	*p* < 0.05 *
	E, CL, LEV	1/18	0.06	7/13	0.54	19.83 (2.00—196.39)	*p* < 0.01 **

Key terms: AMX, amoxicillin; AK, amikacin; CAZ, ceftazidime; CF, cefalotin; CIP, ciprofloxacin; CL, clindamycin; CRO, ceftriaxone; E, erythromycin; ETP, ertapenem; FOS, fosfomycin; GEN, gentamicin; LEV, levofloxacin; MRP, meropenem; OXI, oxacillin; SAM, ampicillin/sulbactam; SXT, trimethoprim/sulfamethoxazole. Data are presented as absolute numbers and percentages (*n*/N, %) of isolates exhibiting simultaneous resistance to all antimicrobial agents included in each predefined combination. Differences in resistance profiles between outpatient and inpatient groups were evaluated using Pearson’s chi-square test or Fisher’s exact test (for cells with expected frequencies < 5). Odds ratios (OR) with corresponding 95% confidence intervals were calculated to measure the association strength between inpatient isolates and resistance profiles. A two-sided *p*-value < 0.05 was considered statistically significant (* *p* < 0.05, ** *p* < 0.01, *** *p* < 0.001). N/A, not applicable; NS, not significant.

## Data Availability

The original contributions of this study are included in the article. Further inquiries can be directed to the corresponding authors.

## Abbreviations

The following abbreviations are used in this manuscript:

AMR	Antimicrobial resistance
UTIs	Urinary tract infections
IMSS	Instituto Mexicano del Seguro Social
MDR	Multidrug resistance
CMN	Centro Médico Nacional
AMP	Ampicillin
AMX	Amoxicillin
AK	Amikacin
CAZ	Ceftazidime
CF	Cefalotin
CIP	Ciprofloxacin
CL	Clindamycin
CRO	Ceftriaxone
CXT	Cefotaxime
CXMA	Cefuroxime axetil
CXM	Cefuroxime
DAP	Daptomycin
DO	Doxycycline
DOR	Doripenem
E	Erythromycin
ETP	Ertapenem
FEF	Cefepime
FOS	Fosfomycin
NIT	Nitrofurantoin
GEN	Gentamicin
LEV	Levofloxacin
LNZ	Linezolid
MRP	Meropenem
MXF	Moxifloxacin
NOR	Norfloxacin
OXI	Oxacillin
RA	Rifampicin
SAM	Ampicillin/Sulbactam
SXT	Trimethoprim/Sulfamethoxazole
TGC	Tigecycline
TE	Tetracycline
VA	Vancomycin
